# Transforming waste management in an eye hospital in Nepal

**Published:** 2021-07-20

**Authors:** Sanjay Kumar Singh

**Affiliations:** 1 Ophthalmologist and Director of the Eastern Regional Eye Care Programme: Biratnagar Eye Hospital, Biratnagar, Nepal.

**A hospital in Nepal has transformed the way it manages waste and is now aiming for zero-waste status, thereby improving the safety of patients, staff members, and the community.**

Health care waste management is a major challenge in all eye hospitals. Hazardous hospital waste has a negative impact on the health of patients, health workers, and the local community. It is believed that 80–85% of eye hospital waste is non-hazardous and, if segregated properly at source, can be treated safely under normal conditions. It is the remaining 15–20% of hazardous waste for which proper disposal is challenging.

Hazardous waste is produced in the course of health care activities such as patient examination, diagnosis, and treatment. Examples from an eye hospital setting include used cotton swabs, eye pads, body tissue and fluids from ocular surgery, and sharps (needles, scalpels, wires, etc.). Non-hazardous waste is generated during administrative work, in the canteen, during cleaning/housekeeping, during construction and demolition, and when carrying out routine maintenance of hospital infrastructures.

**“The lack of a safe waste disposal system increased the risk of infection and health related concerns.”**

Here we share our experience at Biratnagar Eye Hospital regarding safe waste disposal practices in a busy eye hospital setting. Biratnagar Eye Hospital is a high-volume eye hospital providing high quality yet affordable eye care services in the eastern part of Nepal. In 2019, 300,262 patients visited the outpatient department and 70,550 operations were performed.

**Figure:**
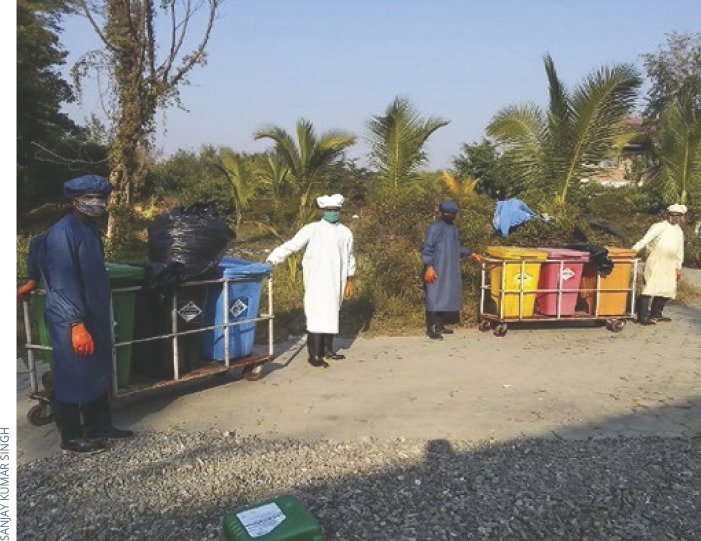
Waste bins are collected several times a day and transported to collection sites in closed bins. **NEPAL**

## Where we started

Prior to the implementation of safe handling and disposal practices at Biratnagar Eye Hospital, hospital waste was collected, without any segregation at source, by untrained staff members who were not protected with the appropriate PPE. This mixed waste was then transported, in an open rickshaw, to a holding area. Because the waste was not separated, the hazardous waste contaminated the non-hazardous waste, so that all the waste was now considered hazardous, and this meant that the overall volume of hazardous waste increased.

The waste was incinerated at irregular intervals in the hospital incinerator, or was removed every 1–2 weeks by the municipality waste collection service.

The local municipality does not segregate waste; waste collected from hospitals and clinics is mixed in with household and industrial waste collected from different sources. All waste is dumped at the same municipality site. The lack of a safe waste disposal system increased the risk of infection and health related concerns to housekeeping staff members, patients, and the general public/community.

## An improved system

Recognising the need to change towards safe waste disposal at Biratnagar Eye Hospital, a policy of health care waste management and standard operating procedures was formulated, taking into consideration national^1^ and international^2^ guidelines. A plan was prepared with the help of a hospital waste management consultant. We wanted to move towards achieving “zero waste” and a commitment was made to ensure that no hazardous waste would be transported out of the hospital premises without proper sterilisation. The plan included guidelines or the segregation, storage, treatment, and disposal of waste.

### Waste segregation

A colour coding system as per guidelines from WHO was introduced. Bins placed in areas accessed by patients and visitors were accompanied by posters explaining good waste segregation practice. Needle destroyer machines have been installed within each clinic, and the main hospital storage area has separate collection chambers to enable correct segregation.

### Staff training

Hospital staff members have been trained in proper waste segregation and disposal. Housekeeping staff who handle waste receive training in the appropriate use of personal protective equipment (PPE) and the safe handling, transportation, and treatment of waste.

As per WHO recommendations, the following PPE is used:

- Heavy-duty utility gloves
- Reusable plastic aprons (cleaned with soap and water, and then decontaminated with 0.5% sodium hypochlorite solution after each use)
- Single-use gloves made of nitrile or latex
- Gowns, which are discarded as infectious waste after each use (and not reused).

Hand hygiene is performed before donning and following removal of PPE.

All housekeeping staff have been immunised against hepatitis B and tetanus.

### Transportation of waste

Following segregation, hazardous waste and non-hazardous waste is collected and transported, in closed bins, to collection sites several times as day, as required.

### Treatment of waste

Infectious waste is sterilised in the waste storage area using an autoclave or sodium hypochlorite solution.

Biodegradable three-chamber pits were constructed for the storage and treatment of non-infectious biodegradable waste (such as food). After three months, biodegradable waste is converted into manure and is used as a compost for the plants in the hospital grounds.

### Refresher training and regular monitoring

Newly recruited staff receive training as part of their induction and refresher training is provided for all staff as part of our regular hospital activities. Routine monitoring of the hospital waste management process is carried out daily by the HCW/HWM consultant. Reports are shared with the hospital waste management committee on a regular basis and improvements actioned as necessary.

### Waste water and faecal waste management

Proper management of waste water (collected from different service) and faecal waste are significant challenges. Each day, around 100,000 litres of waste water and fecal waste are produced at Biratnagar Eye Hospital. We have established a Decentralized Waste Water Treatment System (DEWATS) which is designed to utilise environmental bacteria, plants, and gravity. DEWATS comprises different modules, ranging from settler to superior anaerobic systems such as baffle reactors, fixed-bed filters, and aerobic systems such as a planted gravel filter and collection chamber. When collected, DEWATS-processed water can be repurposed to irrigate farmland surrounding the hospital. Excess treated water is discharged into the public drainage system.

**Figure:**
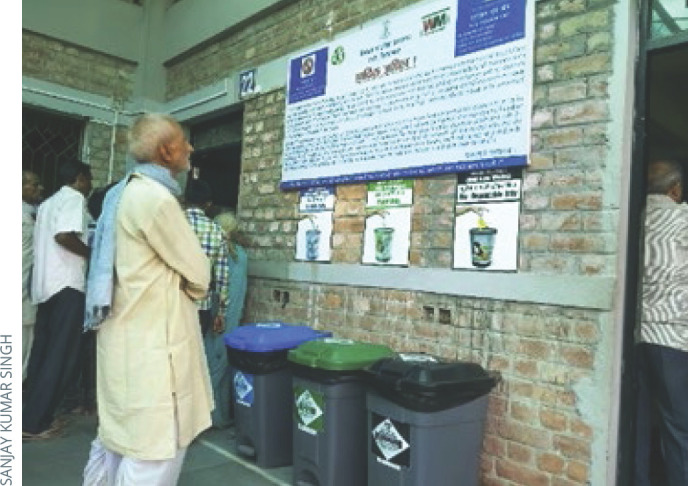
Bins are accompanied by posters explaining good waste segregation practices. **NEPAL**

**How the new waste disposal system was planned and implemented**

We took the following steps:

1. Forming a Hospital Waste Management Committee
2. Formulating policies and standard operating procedures for safe health care waste management
3. Training all staff members in health care waste management
4. Immunising all staff members
5. Purchasing new equipment and setting up storage areas as well as treatment and disposal facilities
6. Starting segregation, storage, treatment, and disposal activities
7. Monitoring the system and offering continuous support to achieve zero waste.

### Environment-friendly equipment and instruments

Our hospital has adapted a policy of using environmentally friendly instruments and equipment wherever possible. For example, we have replaced mercury thermometers with digital thermometers, and replaced fluorescent tube lighting with long-lasting compact fluorescent light bulbs.

### Zero waste and ISO accreditation

By the end of 2019, no hazardous waste was transported out of the hospital without proper treatment. During the year 2018 and 2019, a total of USD $1,889 was generated by the selling of sterilized waste (glass, bottles, and paper). In 2020, the hospital was accredited by ISO 14001:2015 in recognition of the organisation’s Environment Management System.

**Enabling factors in the implementation of a safe waste disposal system**

- A change in the attitude of, and continuous commitment by, the management team
- Identification of a ‘champion’ – a staff member who is interested and can take a lead
- Staff member involvement in the planning and implementation process
- Continuous training and refresher training of staff members
- Strengthening of the monitoring and feedback mechanism

The implementation of an environmentally friendly and safe health care waste management system at Biratnagar Eye Hospital was made possible because of a change in attitude and commitment from the management team, supportive leadership, and the involvement of many staff members.
